# Address Read before the Northern Ohio Dental Association

**Published:** 1871-07

**Authors:** E. J. Robinson

**Affiliations:** President


### ADDRESS READ BEFORE THE NORTHERN OHIO DENTAL ASSOCIATION.

At Put in Bay, May 2, 1871.

BY E. J. ROBINSON, PRESIDENT.

GENTLEMEN: Here, on this beautiful island, made historic by the immortal Perry, and his brave comrades, we have been permitted to assemble at our annual meeting, to enjoy the pleasures of social intercourse, and for that interchange of thought so essential to all who would succeed in our noble profession.

To me, these meetings have always been attended with great pleasure, and no small profit, and I hope this association will continue its labors until it has under its fostering care every reputable dentist in Northern Ohio. Next to our dental colleges, our associations have been the means of elevating the profession to its present standard, and may we not hope by renewed effort to achieve still greater success? No man among us is so humble or obscure, that he may not have it in his power to assist his fellow laborer in a greater or less degree, and I here express the hope that, in the future, as in the present gathering, discussion of the several subjects presented will be participated in by the members generally. We can not all be orators or even fluent speakers, but we can express ourselves in terms that will be understood, and perhaps *felt*, by our more gifted brethren.

There is hardly one among us who does not feel himself a better man and better dentist after having partaken of the feast offered at our annual gatherings. Here we have the beauties of dentistry freed from the perplexities of our daily practice—and truly, dentistry is a beautiful art. To fully appreciate its beauties, we must by diligent and well directed study, educate ourselves to a proper execution of those finer points, which can only be attained by years of patient toil.

Perhaps there are no persons in the community more directly connected with human beauty, which is the highest form of beauty, than the dentist; for the most beautiful and expressive part of the face is the mouth, and the expression of the mouth is dependent upon the teeth, and the teeth of the present generation are largely dependent upon the dentist. In vain will the eyes sparkle with joy and delight if the lips are compressed to hide a mouth full of defective teeth. The whole countenance, beaming with brightness, loses half its charm by the exhibition of a foul and unsightly denture. Half the charms of real culture are lost when expressed through an unsightly denture, and the expression of sorrow and grief is made hideous by the exhibition of this living tomb of decay. It will be seen, therefore, that the function of the dentist is to assist nature to bring forth her best points, to bring out "whatsoever things are pure, whatsoever things are lovely," by removing all the obstructions and arresting all the decays, regulating all the deformities and assisting her to do what she oftentimes in vain endeavors to perform.

But how to do this great work there can never be given any prescribed and invariable rules. We can all profit by the experience and genius of our predecessors, and return our thanks to the laborers that have gone before, but the duties of to-day belong to us alone, and we alone are responsible for the faithful performance of this sacred trust committed to our charge.

And now, gentlemen, how shall we best meet the responsibilities we have voluntarily imposed upon ourselves? There has been so much written and so much said upon the subject, that it is very doubtful whether I shall be able to advance a single thought that has not in some manner presented itself before. Still I am unwilling to let the present opportunity pass without some few suggestions.

There may be, and no doubt are, individuals who in their own estimation are so learned that they can dispense with the aid of our associations, and I shall be doing no injustice to such, if I say that no one knows everything relating to our profession, and he must be a very dull scholar who can not glean some valuable information from even an inferior mind.

Coming together as we do, each from his separate practice, with some special line of thought before him, it is but fair to presume that we may be able to add something to the general fund and thereby be individually benefited. Another great object we should ever have in view, is the public good. The great end of life is not self aggrandizement; he, therefore, who endeavors to benefit self alone, will find himself so narrowed in his thoughts and actions, as to be incapable of success, even in the direction he himself has marked out, and will ere long become a moral leper, shunned by all, and thus it will ever be.

In my intercourse with the profession, I have always found that he who, by a diligent study of dentistry and its kindred sciences, labors hardest to elevate our standard, finds himself most benefited by its ennobling influence, and before he himself is well aware of how it has been brought about, is standing at the head of the profession.

Let us then, each and every one of us, during the interval between our meetings, so apply himself that he can when we meet again, contribute his mite to the advancement of dental science, and by so doing, elevate himself and assist in making the meetings of our Northern Ohio Dental Association pleasant and instructive; a benefit to the patient as well as to the operator.

Gentlemen of the association, I thank you for the honor you have conferred upon me by placing me at the head of your honorable body, and for the kindness and leniency shown me in my short comings, well knowing that it is by your generous forbearance I have been enabled to perform the duties even in a very imperfect manner.

Again, thanking you, I wish you God speed in all your laudable undertakings.

